# Mechanical Properties and Microstructure of Rice Husk Ash–Rubber–Fiber Concrete under Hygrothermal Environment

**DOI:** 10.3390/polym15112415

**Published:** 2023-05-23

**Authors:** Heng Wang, Jianyong Pang, Yihua Xu

**Affiliations:** School of Civil Engineering and Architecture, Anhui University of Science and Technology, Huainan 232001, China; 18451166036@163.com (H.W.);

**Keywords:** hot and humid environment, orthogonal test, dry–wet cycle, microscopic, anti-fatigue performance

## Abstract

In order to study the mechanical properties of rice husk ash–rubber–fiber concrete (RRFC) under hygrothermal environment, the optimal group was selected by orthogonal test. The mass loss, relative dynamic elastic modulus analysis, strength analysis, degradation degree analysis after cyclic loading and internal microstructure analysis of the optimal group of RRFC samples after dry–wet cycles under different environments and temperatures were compared and analyzed. The results show that the large specific surface area of rice husk ash optimizes the particle size distribution of RRFC specimens, reacts to form C-S-H gel, enhances the compactness of concrete, and forms a dense structure as a whole. The presence of rubber particles and PVA fibers effectively improves the mechanical properties and fatigue resistance of RRFC. The comprehensive mechanical properties of RRFC with rubber particle size of 1–3 mm, PVA fiber content of 1.2 kg·m^−3^ and rice husk ash content of 15% are the best. The compressive strength of the specimens after dry–wet cycles in different environments generally increased first and then decreased, reaching a peak at the seventh dry–wet cycle, and the compressive strength of the specimens under chloride salt solution decreased more than that under clear water solution. Thes provided new concrete materials for the construction of highways and tunnels in coastal areas. Under the premise of ensuring the strength and durability of concrete, it is of great practical significance to explore new roads for energy conservation and emission reduction.

## 1. Introduction

With the concept of green and sustainable development [1], waste recycling is widely used [2,3]. The use of waste automobile tires to make rubber particles into concrete has become an important research topic [4,5,6]. Waste rubber tires not only cause economic waste, but also the accumulation of rubber has a serious impact on the surrounding environment. Adding it to concrete can improve the shock resistance and impact resistance [7,8], durability [9], ductility [10], and freeze–thaw resistance [11]. Rice husk is the main by-product of rice processing [12]. Rice husk can absorb SiO_2_ and many beneficial ions in soil and provide a large amount of amorphous SiO_2_ for human beings [13,14,15]. Many places are treated by incineration or dumping, resulting in waste of resources and environmental pollution [16]. The high temperature calcination of rice husk into rice husk ash is added to concrete to make rubber concrete, which has a strong strengthening and modification effect on cement-based materials, which significantly improves the mechanical properties and durability of concrete [17,18,19].

Due to the shortcomings of self-weight and low tensile strength of concrete, many scholars have made up for these shortcomings by adding various fibers [20,21,22]. Jianqiang Wei et al. [23] found that when the substitution rate of rice husk ash was 30%, the alkaline degradation and mineralization of sisal fiber in cement matrix were alleviated most effectively, which ensured that sisal fiber-made green natural fiber-reinforced cement composites have better tensile properties and durability. S. A. N. Mohamed et al. [24] found that the material showed viscoelastic behavior after adding natural fibers to the composite, and the energy loss was manifested as matrix cracks and broken fibers. Santhosh et al. [25] studied the preparation of hybrid dogwood/rice husk reinforced biocomposites (PREHC) by compression molding technology. Compared with unfilled fiber pseudo dogwood/epoxy resin composites (PJEC), rice husk can improve the mechanical properties of PREHC by up to 45%. Muflikhu et al. [26] found that the digital microscope and optical microscope had a good evaluation effect on the new hybrid laminates in the tensile test, but for the bending test, in addition to the optical microscope, it is recommended to use SEM for further analysis to evaluate. Vitale et al. [27] investigated the mechanical properties and failure modes of fiber-reinforced sandwich panels under three-point bending, and established an analysis model to predict the response of different panel–core combinations. Rajak et al. [28] investigated the progress of synthetic fibers in various reinforced composites, considering compatibility, adhesion, excellent performance, optimal orientation and suitable shape. Organic and inorganic fibers were discussed, with emphasis on characteristics, failure modes and applications. Polyvinyl alcohol (PVA) fiber has excellent crack resistance. It is made of high-quality PVA with high degree of polymerization. It is widely used in the field of concrete engineering [29,30,31]. The incorporation of PVA fiber into rice husk ash rubber concrete makes the concrete have higher elastic modulus, greater tensile strength, aging resistance and alkali resistance [32,33]. Therefore, adding PVA fiber to rice husk ash rubber concrete can improve the mechanical properties of rubber concrete, improve its ductility, and improve the strength reduction caused by the addition of rubber. It has a certain improvement effect on rubber concrete, so that it can meet the requirements of new building materials [34].

Although rubber concrete has many advantages such as low density, high energy consumption and good toughness, previous research results show that the weakening effect of rubber particles on concrete strength cannot be ignored, which has indeed become a bottleneck restricting the promotion and application of rubber concrete. Therefore, this paper also considers that the building structures such as highways and tunnels in coastal areas are seriously affected by hot and humid environment [35], chlorine salt erosion [36] and cyclic load [37]. Rice husk ash is used to improve the strength of concrete, and PVA fiber is used to improve the toughness and corrosion resistance of concrete. The three play a role in different damage stages, produce synergistic effects, achieve the purpose of strengthening and toughening in hot and humid environment, and mitigate such hazards. Therefore, this paper analyzes the mechanical properties of concrete with different rubber particle size, different rice husk ash content and different PVA fiber content, and selects the optimal group through orthogonal test [38,39]. The mechanical properties of chloride corrosion resistance in hot and humid environment and the damage characteristics under cyclic loading and unloading are studied.

## 2. Test

### 2.1. Raw Materials

**Figure 1:**
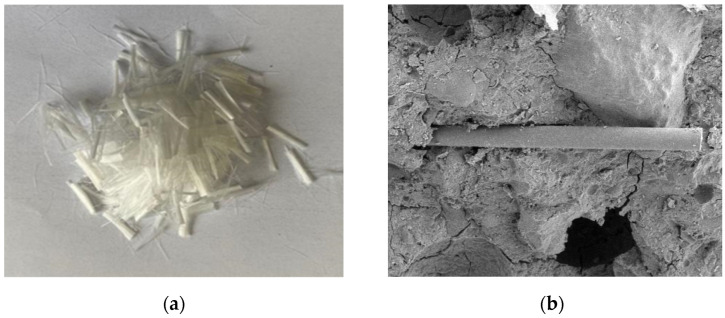
PVA fiber. (**a**) PVA fiber physical map (1:1); (**b**) Micrograph of PVA fiber (50 μm).

The cement used in the test is P·O 42.5 ordinary Portland cement produced by the Bagongshan Cement Plant in Huainan City; the rubber particles cut and crushed from waste tires were selected, and the average particle sizes were 0.4–0.6 mm, 1–2 mm and 2–4 mm, respectively. The rice husk ash is grade I fly ash produced by the Huainan Pingwei Power Plant. The main chemical composition is shown in Table 1. Coarse aggregate uses stone with particle size of 5–15 mm; the fine aggregate is medium sand collected from the Huaihe River in China; the polyvinyl alcohol fibers used in this paper are obtained from Shanghai Film Industry Development Co., Ltd. (Shanghai, China), with lengths of 10 mm, 12 mm and 14 mm, respectively. The performance indicators are shown in Table 2, physical and microscopic diagrams are shown in Figure 1. The water-reducing agent is HPWR standard high-performance water-reducing agent. Mixing water uses ordinary tap water.

### 2.2. Test Scheme Design

**Figure 2:**
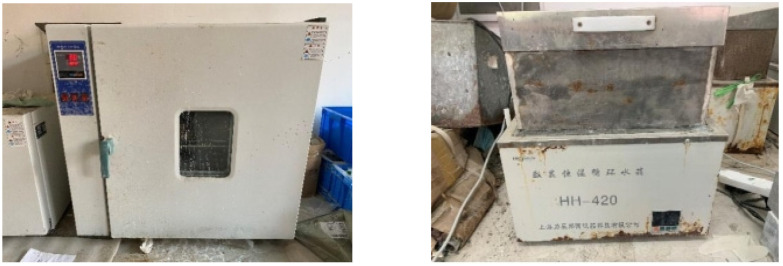
RRFC dry–wet cycle process. (**left**) Electric tachometer indicator thermostatic drying oven; (**right**) Constant temperature circulating water tank.

In this paper, aiming at examining the two problems of solid waste recycling and fiber-reinforced concrete characteristics, the rice husk ash–rubber–fiber concrete is studied. The main research contents are as follows:

(1) According to the standard design C30 ordinary concrete mix, efore the test block is made, the concrete wet mixer with the reference mix ratio is first used to reduce the test error. During the production, the pre-weighed coarse aggregate, fine aggregate, rubber particles, rice husk ash and PVA fiber were mixed and stirred for 2 min, then cement was added and stirred for 1 min, and then the uniformly mixed water and water reducer were injected into the mixer and stirred for 3 min. In order to prevent the rubber floating, the concrete was poured into the mold in three rounds and placed on the vibration table to vibrate and compact until no bubbles were generated, and then the surface was flattened. Then, the concrete was cured in a curing room with a temperature of (20 ± 3) °C and a relative humidity of 70%. After 24 h of curing, the specimens were removed and then placed in a saturated Ca(OH)_2_ solution at (20 ± 2) °C for 28 days.

(2) The rubber particle size (factor A), rice husk ash content (factor B) and PVA fiber (factor C) were set as the three factors of the test, and each factor was set at three levels. The three-factor three-level orthogonal test was carried out, and the test results were analyzed by the efficacy coefficient method to obtain the best factor–level combination. The orthogonal test factors–levels are listed in Table 3. According to the factor–level in the orthogonal test, the L_9_(3^3^) orthogonal test table was finally selected, see Table 4. In the experiment, rubber, PVA fiber and rice husk ash were mixed into concrete to configure a new type of rubber shotcrete. The mix ratio is shown in Table 5.

(3) According to the optimal mix ratio obtained by the orthogonal test, a cylindrical rice husk ash–rubber–fiber concrete test block with a diameter of 50 mm and a height of 100 mm was prepared. After grinding and drying, the dry–wet cycle test was carried out in different environments. The specimens were divided into six groups and placed in a constant temperature water bath at 20 °C, 60 °C and 80 °C, respectively. Two solutions were set for each temperature gradient, including water and a 5% NaCl solution. In order to accurately investigate the influence of hygrothermal environment on the fatigue performance of RC, the dry–wet cycle test (soaking 16 h + drying 6 h + cooling 2 h) was used to simulate the hygrothermal environment under laboratory conditions. The number of dry–wet cycles was 0, 7, 14, 28 and 56, respectively. The dry–wet cycle test process is shown in Figure 2. Then, the quality of RRFC in different dry–wet cycle stages was weighed and recorded. Finally, the compressive test and cyclic loading and unloading test were carried out on the damaged specimens to study the mechanical properties of chloride corrosion resistance in hot and humid environment and the damage characteristics after constant amplitude cyclic loading and unloading. The calculation formulas of stress and strain of uniaxial compressive strength are Equations (1) and (2).

According to the standard for test methods of physical and mechanical properties of concrete (GB/T50081-2019), the calculation formula of uniaxial compressive strength stress of RRFC cylinder is as follows:

(1) $$\sigma =\frac{N}{A}.$$

In the formula, σ is the uniaxial compressive stress (MPa) of concrete cylinder test block; N is the load applied to the concrete cylinder test block (*N*); A is the bearing area of concrete test block (mm^2^).

The uniaxial compressive strain calculation formula of RRFC cylinder is as follows:

(2) $$\varepsilon =\frac{L-L_{0}}{L}$$

In the formula, *ε* is the uniaxial compressive strain (MPa) of the concrete cylinder test block; *L* is the length of concrete cylinder after deformation (mm); *L*_0_ is the original length of concrete cylindrical test block, *L*_0_ = 100 mm.

(4) The microstructure and material composition of the interface area of the optimal group of concrete aggregate were analyzed. The internal damage of concrete was reflected by non-destructive ultrasonic testing technology [40,41] and scanning electron microscopy, and the reason for resistance to chloride erosion in hot and humid environment was explained, and the mechanism of concrete erosion and deterioration was revealed.

## 3. Test Results and Analysis

### 3.1. Orthogonal Test Results

The mix ratio and strength results of each group of concrete tests are shown in Table 6. According to the test results of Table 6, the satisfactory and allowable values of compressive strength, splitting tensile strength and flexural strength of RRFC are determined, as shown in Table 7.

The efficacy coefficient of each index of RRFC was calculated by using the satisfactory value and the allowable value of each index in Table 7. The calculation formula is as follows:

(3) $$f_{i}=\frac{\chi_{i}-\chi_{i}^{s}}{\chi_{i}^{h}-\chi_{i}^{s}}\times 40+60$$

In the formula, *i* is the evaluation index, *i* = 1,2,3; $f_{i}$ is the efficacy coefficient value of *i* index of RRFC specimen; $\chi_{i}^{s}$ is an impermissible value of index *i*. $\chi_{i}^{h}$ is the satisfactory value of *i* index.

According to the above Formula (3), the efficiency coefficient value is calculated and analyzed, and the results are shown in Table 8.

According to the analysis results of the efficacy coefficient method in Table 8, the total efficacy coefficient of the fifth group was the highest, and the total efficacy coefficient was 93.3, indicating that its performance was the best and better than that of the other mix ratios. Therefore, the fifth group was selected as the best ratio for this experiment. The optimal ratio is A2B2C3, that is, the particle size of rubber particles is 1–3 mm, the content of PVA fiber is 1.2 kg·m^−3^, and the content of rice husk ash is 15%. According to the optimal ratio, concrete test blocks were made and subsequent tests were carried out.

### 3.2. RRFC Mass Loss in Hot and Humid Environment

The quality of RRFC concrete is constantly changing under the double damage of chloride erosion and dry–wet cycle. The mass of concrete before and after the dry–wet cycle test was weighed, and the mass change rate ∆*W_m_* of RRFC test block under different dry–wet cycles was calculated by the following formula:

(4) $$\Delta Wm=\frac{m_{0}-m_{n}}{m_{0}}\times 100\%$$

where ∆*W_m_* is the concrete quality change rate/%; *m*_0_ is the initial mass of concrete test block/kg; *m_n_* is the mass/kg of the concrete test block after n dry–wet cycles.

**Figure 3:**
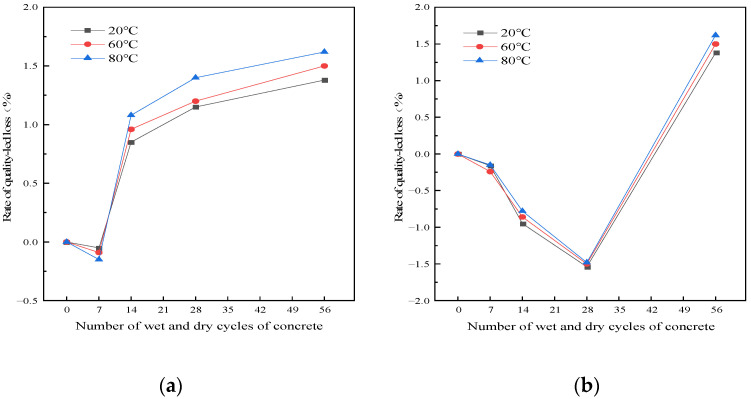
The relationship between RRFC dry–wet cycles and mass loss rate. (**a**) Clear aqueous solution. (**b**) Chloride solution.

The curve of the mass loss rate of RRFC test block with the number of dry–wet cycles in different environments is shown in Figure 3.

It can be seen from Figure 3 that the quality of RRFC specimens increased first and then decreased with the progress of the dry–wet cycle test. In the clear water solution, the mass of the specimen increased slightly at the beginning of the dry–wet cycle, and the mass of the specimen decreased significantly after the seventh dry–wet cycle. This is due to the reaction between SiO_2_ in rice husk ash and Ca(OH)_2_ in the process of cement hydration in the early stage of the dry–wet cycle, which generates a large number of C-S-H gel to fill the holes and increase the quality. With the progress of the dry–wet cycle, water molecules are continuously immersed and baked inside the concrete, and the holes inside the concrete test block are increasing, even penetrating into cracks. The cementation force of the internal structure is weakened, and some internal particles are brought to the external environment, resulting in the continuous reduction in concrete quality. In the chloride salt solution, the mass of the specimen increased with the increase in the number of dry–wet cycles in the early stage of the dry–wet cycle, and the mass of the specimen reached the maximum peak at 28 dry–wet cycles. The reason is that the dry–wet cycle in the salt solution consists of the C-S-H gel produced by rice husk ash and Ca(OH)_2_, and it also contains the impurities and NaCl in the solution that are attached to the internal holes and surfaces of the concrete test block. The chloride salt forms salt crystal erosion damage to the concrete, and the crystallization accumulates in the cracks of the internal holes of the concrete; in this way, the quality of the concrete is increasing.

When the number of dry and wet cycles is the same, the higher the solution temperature, the greater the mass loss rate of the specimen. When the temperature of the clear water solution is 80 °C, the mass loss of the test block is the largest, and the mass loss rate is 1.62%. The temperature of the clear water solution is 60 °C, and the mass loss is the smallest at 20 °C with the mass loss rate of 1.38%. This is because the dry–wet cycle itself has a certain deterioration effect on the concrete specimen, and the increase in the temperature leads to the acceleration of the crystallization erosion diffusion of the chloride salt on the concrete specimen, which accelerates the deterioration rate of the concrete specimen, and the deterioration damage is more serious.

### 3.3. Analysis of Relative Dynamic Elastic Modulus

The relative wave velocity of RRFC was measured by NN-4B non-metallic ultrasonic testing analyzer manufactured by Beijing Kangkerui Company (Beijing, China), and the relative dynamic elastic modulus of concrete specimens was calculated. The calculation formula is as follows:

(5) $$E_{\mathrm{dr}}=\frac{E_{\mathrm{dt}}}{E_{d0}}\times 100\%=\frac{T_{0}^{2}}{T_{t}^{2}}\times 100\%=\frac{V_{t}^{2}}{V_{0}^{2}}\times 100\%$$

where *E_dr_* is relative dynamic elastic modulus/%; *E_dt_* and *E_d_*_0_ are the dynamic elastic modulus/MPa of the specimens after different dry–wet cycles and without dry–wet cycles. *T_t_* and *T*_0_ are the sound time/s of the specimens after different dry–wet cycles and without dry–wet cycles. *V_t_* and *V*_0_ are the ultrasonic wave velocity/m·s^−1^ of the specimens after different dry–wet cycles and without dry–wet cycles.

**Figure 4:**
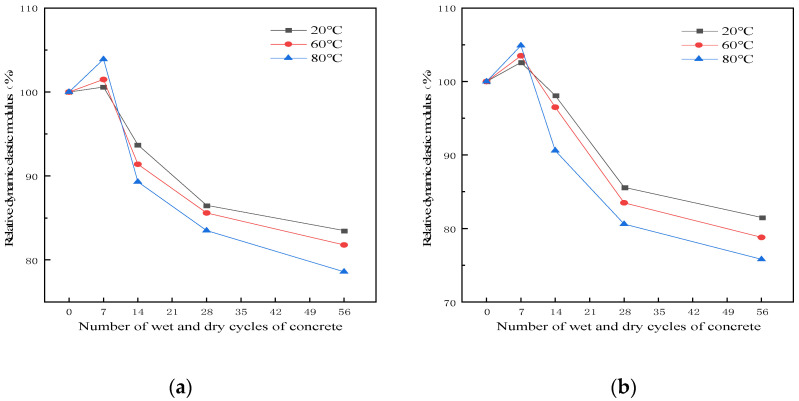
The relationship between the RRFC dry–wet cycles and relative dynamic elastic modulus. (**a**) Clear aqueous solution. (**b**) Chloride solution.

Figure 4 depicts the relationship between the relative dynamic elastic modulus and the number of dry–wet cycles of the RRFC specimens after different dry–wet cycles in aqueous solution and chloride solution at different temperatures.

It can be seen from Figure 4 that with the progress of the dry–wet cycle, the relative dynamic elastic modulus of RRFC specimens in the two solutions at different temperatures generally increased slightly and then decreased significantly. In the chloride solution, the relative dynamic elastic modulus of RRFC specimens increased first and then decreased significantly. Compared with the clear water solution, the peak value of the relative dynamic elastic modulus of the RRFC specimens in the chloride salt solution was relatively higher, and the relative dynamic elastic modulus of the concrete was more moderate than that of the clear water solution in the early stage of the dry–wet cycle. This is due to the dense matter inside the concrete, not only the full and complete hydration of the cement mentioned above, and the existence of C-S-H gel. The chloride salt in the chloride salt solution is eroded by the chloride salt produced during the dry–wet cycle. More chloride salt crystals are accumulated in the gaps and holes inside and on the surface of the concrete, filling the gaps, making the internal structure of the concrete denser, improving the compactness, and making the relative dynamic elastic modulus higher.

It can be seen from Figure 4 that under the same number of dry–wet cycles, the higher the solution temperature, the faster the decrease in relative dynamic elastic modulus of the RRFC specimen, and the more serious the specimen damage. Taking chlorine salt solution as an example, when the temperature is 20 °C and the number of dry–wet cycles is 0, 7, 14, 28 and 56, the relative dynamic elastic modulus is 100%, 102.6%, 98.1%, 85.6% and 81.5%, respectively. When the temperature is 60 °C and the number of dry–wet cycles is 0, 7, 14, 28 and 56, the relative dynamic elastic modulus is 100%, 103.5%, 96.5%, 83.5% and 78.8%, respectively. When the temperature is 80 °C and the number of dry–wet cycles is 0, 7, 14, 28 and 56, the relative dynamic elastic modulus is 100%, 104.9%, 90.6%, 80.6% and 75.8%, respectively.

### 3.4. Strength Analysis

The compressive strength corrosion resistance coefficient *K_f_* of RRFC specimens after different dry–wet cycles is calculated by the following formula:

(6) $$K_{\mathrm{fn}}=\frac{f_{n}}{f_{0}}\times 100,$$

where *K_fn_* is the compressive strength corrosion resistance coefficient/% of concrete specimens after *n* dry–wet cycles; *f_n_* is the compressive strength/MPa of concrete specimens after n dry–wet cycles; *f*_0_ is the compressive strength/MPa of the specimen before the dry–wet cycle.

**Figure 5:**
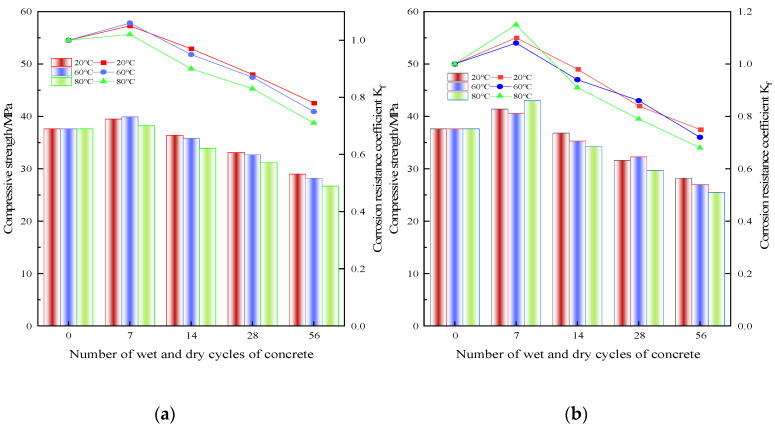
Relationship between dry–wet cycles and corrosion resistance coefficient of RRFC specimens. (**a**) Clear aqueous solution. (**b**) Chloride solution.

Figure 5 shows the curves of compressive strength and corrosion resistance coefficient of the RRFC specimens with the two solutions at different temperatures in the dry–wet cycle mechanism.

It can be seen from Figure 5 that the corrosion resistance coefficient of the RRFC specimens in the two solution environments at different temperatures increases first and then decreases with the increase in dry–wet cycles, but there are some differences in the specific changes.

In the clear water solution, the compressive strength of RRFC specimens increased with the increase in the number of dry–wet cycles and peaked at the seventh time. In the early stage of the dry–wet cycle, the incompletely hydrated cement inside the concrete specimen continues to undergo hydration reaction, so that the hydration reaction is carried out thoroughly, the cementation force is enhanced, and the compressive strength of the specimen is enhanced. The rice husk ash in the specimen has high pozzolanic properties. The highly active SiO_2_ reacts with Ca(OH)_2_ generated during the hydration reaction to form C-S-H gel, which fills holes and gaps, improves the compactness, alleviates the damage caused by the dry–wet cycle process, and improves the compressive strength and corrosion resistance of the RRFC specimen. As the number of dry–wet cycles increases, the compressive strength of the specimen gradually decreases, and the corrosion resistance coefficient continues to decrease. The higher the solution temperature, the greater the compressive strength damage of the specimen, the greater the decrease in the corrosion resistance coefficient, and the lower the residual compressive strength. After 56 dry–wet cycles, the compressive strength of the specimen reached 29 MPa and the corrosion resistance coefficient was 0.78 when the solution temperature was 20 °C. When the solution temperature was 60 °C, the compressive strength of the specimen reached 28.2 MPa, and the corrosion resistance coefficient was 0.75. When the solution temperature was 80 °C, the compressive strength of the specimen reached 26.7 MPa and the corrosion resistance coefficient was 0.71.

In the chloride solution, the compressive strength of RRFC specimens increased with the increase in the number of dry–wet cycles and reached the peak at the seventh time, and the peak compressive strength was significantly greater than that in the clear water solution. This is because in the chloride solution, not only the cement hydration reaction is more thorough and complete, but also the highly active SiO_2_ in the rice husk ash reacts with Ca(OH)_2_ to form C-S-H gel to fill the pores, and the chloride salt forms crystals attached to the surface and inside of the concrete, filling some pores and cracks and improving the compactness. At this time, the tensile strength provided by the PVA fiber in the specimen is greater than the expansion force generated by the chloride salt crystallization, so the compressive strength of the concrete specimen increases and the corrosion resistance coefficient increases. However, with the increase in dry–wet cycles, the compressive strength of the specimens gradually decreased, and the corrosion resistance coefficient continued to decrease. The higher the solution temperature, the greater the compressive strength damage of the specimen, the greater the corrosion resistance coefficient, and the lower the residual compressive strength.

### 3.5. Analysis of Deterioration Degree after Cyclic Loading

#### 3.5.1. Uniaxial Compressive Strength Test

When the RRFC test block reaches the corresponding dry–wet cycle times (0 times, 7 times, 14 times, 28 times and 56 times) in different hot and humid environments, the uniaxial compression test and cyclic loading and unloading test are carried out to analyze the fatigue damage law.

**Figure 6:**
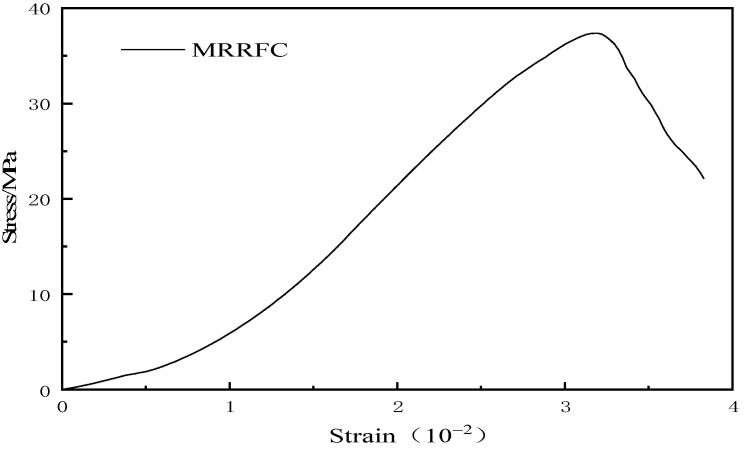
Uniaxial compressive stress–strain curve.

The uniaxial compressive strength stress and strain of the optimal factor level combination are calculated by using Formulas (1) and (2). The uniaxial compressive stress–strain curve is shown in Figure 6.

It can be seen from Figure 6 that the shape of the uniaxial stress–strain full curve diagram of the optimal factor level combination is roughly composed of three parts, namely the linear rise stage, the nonlinear rise stage after the initial crack point and the decline after the peak point. At the initial stage of stress loading, the curve is concave, that is, with the gradual increase in concrete strain, the stress remains at a low level. At this time, with the increase in stress, the internal pores of concrete are gradually compacted and closed, and the specimen tends to be dense, so the stress–strain curve shows a linear upward trend. With the continuous increase in stress, the strain increase rate becomes faster, the curve is convex, the internal pores gradually break and the specimen deforms greatly. After the peak point, the stress–strain curve shows a downward trend, and the stress decreases rapidly with the increase in strain. At this stage, the deformation of the specimen gradually increases, and this continues until the specimen is destroyed.

#### 3.5.2. Cyclic Loading and Unloading Stress–Strain Curve Analysis

**Figure 7:**
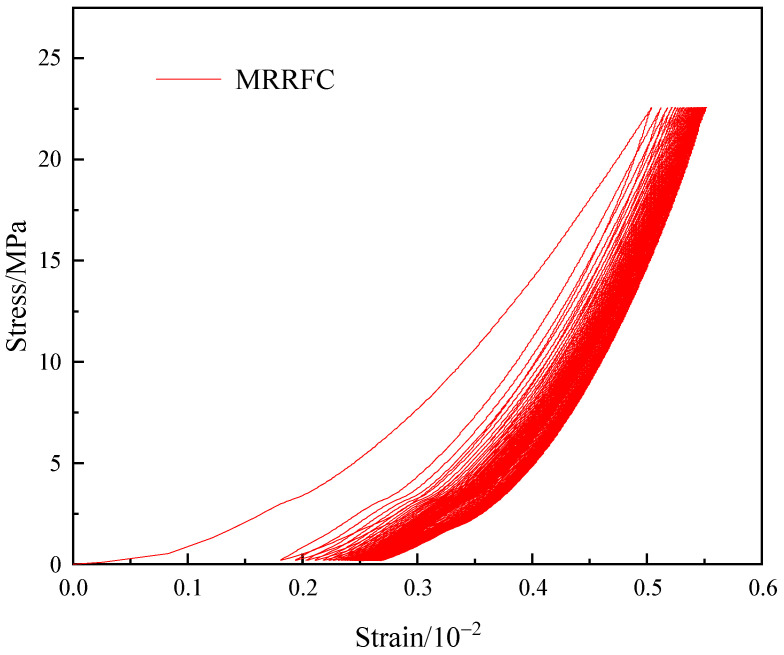
RRFC specimen stress–strain curve diagram.

The cyclic loading and unloading test was carried out on the RRFC specimen after the dry–wet cycle, that is, 60% of the uniaxial compressive failure load of the specimen itself was taken as the upper limit of the loading load, and the lower limit of the unloading load was 100 N. A loading–unloading process was used as a cycle, and a total of 50 cycles was carried out. The test results are transformed into stress and strain by Formulas (1) and (2). Figure 7 is the axial stress–strain curve of RRFC specimen under constant amplitude cyclic loading and unloading.

During the pouring process of concrete specimens, a small amount of pores and cracks inevitably occurs, resulting in the inhomogeneity of concrete materials, and the strength and deformation of different parts of the specimens also shows differences. Therefore, the deformation of RRFC specimens during compression includes reversible elastic deformation and irreversible plastic deformation. The specific situation is also determined by the stress level and the history of unloading and unloading, such as the upper limit of loading stress and the number of cyclic loadings and unloadings. This feature shows that the loading curve and the unloading curve cannot coincide with each other on the stress–strain curve of the specimen, forming a hysteresis loop.

As shown in Figure 7, the stress–strain curve of the RRFC specimen shows two stages of first sparse and then dense. The total strain of the specimen changes little, and the deformation mainly occurs in the first cycle. As the number of cycles increases, the hysteresis loop area of the RRFC specimen decreases, the specimen is dominated by elastic deformation, and the curve tends to be dense. This is because a certain amount of rubber particles is added to the RRFC specimen, and the interface transition zone between the rubber particles and the cement mortar is weak. Therefore, when the test block is subjected to compressive stress at the beginning of the cycle, the pores inside the RRFC specimen quickly penetrate and produce microcracks. At this time, the specimen is mainly subjected to plastic deformation. However, rubber has high elasticity and high toughness, which effectively improves its impact resistance in concrete structures. Therefore, as the number of cycles increases, the specimen is converted into elastic deformation, the hysteresis area decreases continuously, and the curve changes from sparse to dense.

### 3.6. SEM Electron Microscope Test

**Figure 8:**
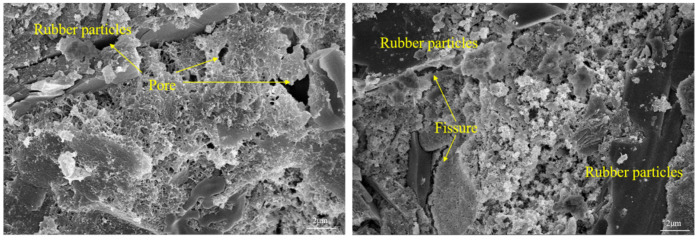
Internal morphology of RRFC specimens under 56 wetting–drying cycles in chloride solution.

Figure 8 shows the internal micro-morphology of RRFC specimens under 56 dry–wet cycles in chloride solution at 80 °C. It can be seen from the diagram that the internal pores and cracks of the specimen increase, and cracks occur at the interface between the rubber particles and the cement matrix. Due to the double damage of the dry–wet cycle and chloride erosion under chloride solution, the specimens with the dry–wet cycle under clear water solution were only damaged by the dry–wet cycle. In the middle and late stages of the dry–wet cycle process, the chloride salt crystallization erosion damage inside the RRFC specimen is serious. The osmotic pressure generated by the flow of water molecules and the expansion force caused by the C_3_A·CaCl_2_·10H_2_O (F salt) generated by the reaction are greater than the tensile strength caused by the polyethylene fiber, resulting in the increase in holes and gradual penetration into gaps. The compressive strength of the specimen decreases significantly and the corrosion resistance coefficient decreases. The higher solution temperature accelerates the thermal movement of chloride ions and water molecules in the solution inside the concrete. The diffusion rate of chloride ions increases and the range becomes wider; the erosion rate increases exponentially, resulting in an increase in the internal solubility of the concrete specimen, a decrease in compactness, and internal structure damage. This explains the reason why the macroscopic mechanical properties of the RRFC specimens began to decrease in the middle of the dry–wet cycle in chloride solution and the decrease was significantly greater than that of the specimens in the clear aqueous solution environment.

**Figure 9:**
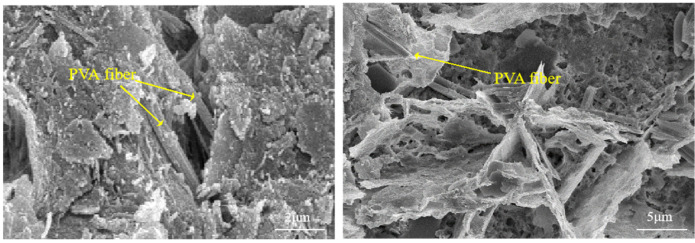
Microstructure of RRFC specimens.

It can be clearly seen from Figure 9 that there are randomly distributed PVA fibers inside the RRFC specimen. As a bridge through cracks and pores, it effectively disperses the end stress of cracks and adjusts the stress redistribution. PVA fibers constitute a random three-dimensional space grid, which effectively hinders the migration channel and crack development of chloride ions. The rubber particles inside the specimen block the crack propagation, passivate the crack, and weaken the stress redistribution of the specimen. This indicates that in order to form a large number of cracks in the RRFC specimen, the expansion pressure generated by multiple dry–wet cycles and the expansion stress provided by the chloride salt crystallization are required. Therefore, the rubber particles and cement matrix are tightly bonded under the bonding of PVA fiber and C-S-H gel, which improves the toughness and corrosion resistance of RRFC specimens.

## 4. Conclusions

Based on the orthogonal test scheme, the feasible ratio combination of new green concrete is explored. At the same time, the dry–wet cycle test and cyclic loading–unloading test of the RRFC specimens under the optimal ratio were carried out, and the damage and deterioration law of the specimens after the dry–wet cycle test was analyzed. The following conclusions were drawn:

(1) The compressive strength of RRFC specimens is between 29.1 MPa and 37.6 MPa, the splitting tensile strength is between 3.2 MPa and 4.5 MPa, and the flexural strength is between 4.6 MPa and 5.8 MPa. Its mechanical properties are mostly better than those of the C30 ordinary concrete, and the maximum increase is 17.5%, 26.7% and 15.7%, respectively. The optimal factor level combination of RRFC specimens is A2B2C3, that is, the rubber particle size is 1–3 mm, the PVA fiber content is 1.2 kg·m^−3^, and the rice husk ash content is 15%.

(2) The best ratio of RRFC specimens was placed in clear water solution and chloride solution at different temperatures for 56 dry–wet cycles. It was found that the compressive strength of the specimens under the dry–wet cycles in different environments increased first and then decreased, and reached the peak in the seventh dry–wet cycle. When the number of dry and wet cycles is the same, the compressive strength of the specimen under the chloride solution is lower than that of the clear water solution, and the higher the solution temperature, the more serious the damage of the specimen. There is a significant correlation between these deterioration damage indexes and the change law of compressive strength of specimens, which proves that the change in mechanical properties of RRFC specimens is closely related to the deterioration damage of specimens under dry–wet cycle erosion.

(3) The cyclic loading–unloading stress–strain curve of RRFC specimen shows two stages of sparse first and then dense. The total strain of the specimen changes little, and the deformation mainly occurs in the first cycle. As the number of cyclic loading–unloading increases, microcracks appear inside the RRFC specimen. At this time, the specimen is dominated by elastic deformation, and the hysteresis loop area decreases. The axial peak strain and residual strain of the RRFC specimen increase continuously during the repeated loading and unloading process. The increase in the axial peak strain in the first five cycles is the most obvious; then, it gradually slows down and tends to be stable, reflecting the trend of the hysteresis curve from sparse to dense.

(4) The large specific surface area of rice husk ash optimizes the particle gradation of RRFC specimens, exerts the filling effect, and makes the interior of the specimens denser. At the same time, a large number of highly active SiO_2_ in rice husk ash reacts with Ca(OH)_2_ to form C-S-H gel, which enhances the compactness of concrete, reduces the concentration of Ca^2+^, promotes the hydration reaction of cement, and continuously forms honeycomb products during the hydration reaction and the surrounding plate-like area increases, so that the specimen forms a dense structure as a whole.

(5) The excellent ductility of rubber particles effectively alleviates the damage caused by the immersion and drying of water molecules and the expansion force caused by chloride crystallization in the dry–wet cycle erosion. In the process of cyclic loading and unloading, the damage caused by repeated loading and unloading is effectively alleviated, and the stress redistribution inside the specimen is optimized, so that RRFC exhibits excellent fatigue resistance. The PVA fiber forms a random three-dimensional space grid in the RRFC specimen, which effectively hinders the migration channel and crack development of chloride ions, alleviates the stress concentration phenomenon, prevents the floating of rubber particles, and improves the mechanical properties and fatigue resistance of the specimen.

(6) Due to the limitation of conditions, only the RRFC specimens with the best ratio were selected for the dry–wet cycle test and cyclic loading–unloading test under different solutions, while the durability and fatigue resistance of RRFC specimens with other dosages need further exploration and research.

## Figures and Tables

**Table 1:** The chemical composition of rice husk ash.

Name	SiO_2_	K_2_O	CaO	Fe_2_O_3_	MgO
content/%	85.6	2.51	2.44	0.56	0.51

**Table 2:** Performance Parameters of PVA Fiber.

Parameter	Diameter/μm	Breaking Strength/MPa	Elastic Modulus/GPa	Breaking Elongation/%	Density/g·cm^−3^
numerical value	15.09	1600–2500	40–80	6	1.29

**Table 3:** Orthogonal test factor–level table.

Factor Level	Factor A Rubber Particle Size/mm	Factor B Rice Husk Ash Content/%	Factor C PVA Content/kg·m^−3^
**1**	0.4–0.6	5	0.6
**2**	1–2	10	1.2
**3**	2–4	15	1.8

Note: The amount of rubber particles is calculated as equal volume replacement, the amount of PVA is calculated according to the volume rate, and the amount of rice husk ash is calculated as equal mass replacement.

**Table 4:** Orthogonal experiment table (L_9_(3^3^)).

Group Number	Factor A	Factor B	Factor C
1	1	1	1
2	1	2	2
3	1	3	3
4	2	1	2
5	2	2	3
6	2	3	1
7	3	1	3
8	3	2	1
9	3	3	2

**Table 5:** Orthogonal test concrete mix proportion.

Number	Sand/kg·m^−3^	Rubber Particle Size (A)/mm	Rice Husk Ash Content (B)/%	PVA Fiber Content (C)/kg·m^−3^
1	617	0.4–0.6	5	0.6
2	617	0.4–0.6	10	1.2
3	617	0.4–0.6	15	1.8
4	617	1–2	10	0.6
5	617	1–2	15	1.2
6	617	1–2	5	1.8
7	617	2–4	15	0.6
8	617	2–4	5	1.2
9	617	2–4	10	1.8

Note: water: 153 kg/m^3^, cement: 373 kg/m^3^, stone: 1121 kg/m^3^, water reducer: 3.5 kg/m^3^.

**Table 6:** Mix proportion and result of orthogonal test.

Number	Cement /kg·m^−3^	Rubber Particle Size (A)/mm	PVA Fiber Content (B) /kg·m^−3^	Rice Husk Ash Content (C) /kg·m^−3^	Compressive Strength /MPa	Split Tensile Strength /MPa	Break-Off Strength /MPa
1	354.35	0.4–0.6	0.6	5	31.6	3.8	5.80
2	335.70	0.4–0.6	1.2	10	34.8	4.2	5.45
3	317.05	0.4–0.6	1.8	15	33.9	4.3	5.52
4	335.70	1–2	0.6	10	37.2	3.6	5.32
5	317.05	1–2	1.2	15	37.6	4.5	5.19
6	354.35	1–2	1.8	5	35.9	4.2	5.52
7	317.05	2–4	0.6	15	29.1	3.2	4.60
8	354.35	2–4	1.2	5	29.6	3.5	5.02
9	335.70	2–4	1.8	10	31.9	3.4	4.78

Note: water: 153 kg/m^3^, sand: 613 kg/m^3^, stone: 1121 kg/m^3^, water reducer: 3.5 kg/m^3^.

**Table 7:** The satisfactory and unacceptable values of each indicator.

	Index	Compressive Strength/MPa	Split Tensile Strength/MPa	Break-Off Strength/MPa
Value Type				
satisfaction value		37.6	4.5	5.8
not allowed value		29.1	3.2	4.6

**Table 8:** Efficacy coefficient analysis results.

Number	Evaluating Indicator			Efficiency Coefficient			Total Efficacy Coefficient Value
	Compressive Strength/MPa	Split Tensile Strength/MPa	Break-Off Strength/MPa	Compressive Strength	Split Tensile Strength	Break-Off Strength	
1	31.6	3.8	5.80	71.76	78.46	100	82.24
2	34.8	4.2	5.45	86.82	90.77	88.33	88.46
3	33.9	4.3	5.52	82.59	93.85	90.67	88.39
4	37.2	3.6	5.32	98.12	72.31	84	86.14
5	37.6	4.5	5.19	100	100	79.67	93.30
6	35.9	4.2	5.52	92.00	90.77	90.67	91.23
7	29.1	3.2	4.60	60	60	60	60
8	29.6	3.5	5.02	62.35	69.23	74	66.51
9	31.9	3.4	4.78	73.18	66.15	66	68.92

## Data Availability

The data used to support the findings of this study are available from the corresponding author upon request.
